# Powder Pressed Cuprous Iodide (CuI) as A Hole Transporting Material for Perovskite Solar Cells

**DOI:** 10.3390/ma12132037

**Published:** 2019-06-26

**Authors:** Siva Uthayaraj, D. G. B. C. Karunarathne, G. R. A. Kumara, Thanihaichelvan Murugathas, Shivatharsiny Rasalingam, R. M. G. Rajapakse, Punniamoorthy Ravirajan, Dhayalan Velauthapillai

**Affiliations:** 1 Department of Physics, University of Jaffna, Jaffna 40000, Sri Lanka; 2 Faculty of Engineering and Science, Western Norway University of Applied Sciences, P.O. Box 7030, 5020 Bergen, Norway; 3 National Institute of Fundamental Studies, Hantana Road, Kandy 20000, Sri Lanka; 4 Department of Chemistry, University of Jaffna, Jaffna 40000, Sri Lanka; 5 Department of Chemistry, Faculty of Science, University of Peradeniya, Peradeniya 20400, Sri Lanka

**Keywords:** perovskite solar cells, hole-transporting material, powder pressing, cuprous iodide, CuI, spiro-OMeTAD, air stable

## Abstract

This study focuses on employing cuprous iodide (CuI) as a hole-transporting material (HTM) in fabricating highly efficient perovskite solar cells (PSCs). The PSCs were made in air with either CuI or 2,2′,7,7′-Tetrakis[N,N-di(4-methoxyphenyl)amino]-9,9′-spirobifluorene (spiro-OMeTAD) as HTMs. A simple and novel pressing method was employed for incorporating CuI powder layer between perovskite layer and Pt top-contact to fabricate devices with CuI, while spiro-OMeTAD was spin-coated between perovskite layer and thermally evaporated Au top-contact to fabricate devices with spiro-OMeTAD. Under illuminations of 100 mW/cm^2^ with an air mass (AM) 1.5 filter in air, the average short-circuit current density (J_SC_) of the CuI devices was over 24 mA/cm^2^, which is marginally higher than that of spiro-OMeTAD devices. Higher J_SC_ of the CuI devices can be attributed to high hole-mobility of CuI that minimizes the electron-hole recombination. However, the average power conversion efficiency (PCE) of the CuI devices were lower than that of spiro-OMeTAD devices due to slightly lower open-circuit voltage (V_OC_) and fill factor (FF). This is probably due to surface roughness of CuI powder. However, optimized devices with solvent-free powder pressed CuI as HTM show a promising efficiency of over 8.0 % under illuminations of 1 sun (100 mW/cm^2^) with an air mass 1.5 filter in air, which is the highest among the reported efficiency values for PSCs fabricated in an open environment with CuI as HTM.

## 1. Introduction

Perovskite solar cells based on methylammonium lead halides perovskite material with spiro-OMeTAD as hole-collector have attracted considerable attention of research and industrial community due to the rapid growth of efficiency from 3.8% to 24.2% [1,2,3,4,5] in recent years. However, spiro-OMeTAD in its pristine form still shows some intrinsic drawbacks, such as relatively low hole mobility and conductivity, as well as it is a highly expensive material and it needs dopants [6]. As such, replacing this hole-collector by a low-cost, dopant-free alternative would result in bringing down the overall cost of the device. Several works have been reported on use of cuprous iodide (CuI) as hole transporting material in place of liquid electrolyte in Grätzel type of solar cells [7,8,9,10,11] as CuI possesses several unique characteristics, such as high transparency, low-production cost, ease of deposition, high hole-mobility, and good chemical stability [12]. With the help of crystal growth inhibitor triethylammonium thiocyanate, over 5.0% efficiency was achieved for all solid-state dye-sensitized solar cells based on CuI HTMs [13,14]. Christians et al. reported the best efficiency of 6.0% with the perovskite solar cell based on CuI HTM (J_SC_ = 17.8 mA/cm^2^, V_OC_ = 0.55 V and FF = 0.62) [15]. In these studies, CuI was chemically deposited using di-n-propyl sulfide and chlorobenzene. Qin et al. used solution processed cuprous thiocyanate (CuSCN) as HTM in perovskite solar cell and obtained an impressive efficiency of over 12.4% [16] under 1 Sun illumination. Huangfu et al. obtained 5.8% efficiency in the perovskite solar cell with spray coated CuI as the HTM [17]. Hu et al. were able to obtain 9.12% efficiency by modifying the photoanode to have TiO_2_ nanotube/TiO_2_ nanoparticle hybrid structure [18] with spray coated CuI. Sepalage et al. obtained an efficiency of 7.5% for perovskite solar cell based on CuI hole transporting material, where the CuI layer was deposited by doctor blading method [19].

CuI has several important properties contributing to its application as an HTM in perovskite solar cells [20]. Takahasi and Suzuki reported that valence band edge of chemically stable CuI is very close to highest occupied molecular orbital (HOMO) level of widely studied spiro-OMeTAD (5.2 eV) hole-transporting materials for PSCs [21,22,23]. Hole mobilities in spiro-OMeTAD and CuI are ~ 2 × 10^−4^ cm^2^V^−1^s^−1^ and 43.9 cm^2^V^−1^s^−1^ respectively [24,25,26,27], which means that the hole mobility of CuI is over 10^5^-fold higher than that of spiro-OMeTAD. CuI thin films can be deposited at low temperature [28] and this film is a p-type semiconductor with a relatively wide band gap of 3.1 eV. Moreover, the cost of CuI is over 10-fold less than that of spiro-OMeTAD, and it can readily be deposited on perovskite film for large scale production of perovskite solar modules. Although CuI seems to have such a vast advantage over spiro-OMeTAD and various techniques have already been adopted for deposition of CuI [3,29] in fabricating PSCs, the highest efficiency values reported for CuI devices is 10% which is 2.2 times lower than that of spiro-OMeTAD devices [30] under a controlled environment. In this reported work [30], a mixed solvent system comprising of chlorobenzene, acetonitrile, and 4-tert-Butylpyridine in 40:20:1 volume ratio has been used, since the pure acetonitrile solvent dissolves the perovskite layer. However, chlorobenzene is a solvent categorized as a toxic substance which has shown acute and intermediate exposure in animals tend to cause narcosis, restlessness, tremors and muscle spasms.

This work attempted to fabricate solvent-free route to make CuI hole-transporting materials by simply pressing CuI between perovskite layer and Pt-coated FTO glass. All the fabrication and characterization processes were carried out in air. This solvent-free pressed method of hole transport material is hazard free and simple method capable for the large-scale production of PSCs. For comparison purpose, PSCs were fabricated by spin-coating spiro-OMeTAD between perovskite layer and thermally evaporated Au top-contact under the same open environment. 

## 2. Materials and Methods

### 2.1. Materials

Fluorine-doped Tin Oxide (FTO, surface resistivity of 10 Ω/square), Methylammonium Iodide (MAI, 98%), Lead (II) Chloride (98%), N,N-Dimethylformamide, (DMF, Anhydrous 99.8%), 2,2′,7,7′-Tetrakis[N,N-di(4-methoxyphenyl)amino]-9,9′-spirobifluorene, spiro-OMeTAD (99%), Chlorobenzene (Anhydrous 99.8%), Bis(trifluoromethane) sulfonamide lithium salt (Li-TFSI, 99.95%), 4-tert-Butylpyridine (96%), Acetonitrile (99.8%), Cuprous Iodide (CuI, 98%), Titanium di-isopropoxide bis (acetylacetonate) 75% (w/w), Ethanol (absolute, for HPLC, ≥99.8%) and poly(3-hexylthiophene-2,5-diyl) were purchased from Sigma-Aldrich (Oslo, Norway). 18NR-T Transparent Titania Paste was purchased from Great Cell Solar (Queanbeyan, Australia). All solvents were used without any further purification.

### 2.2. Solar Cell Fabrication

FTO substrates were cleaned with detergent for 20 min. and then with distilled water for 20 min. and finally, with ethanol for 20 min. in an ultrasonic bath. A dense layer and porous layer of TiO_2_ was deposited by spray and spin coating as reported elsewhere [31,32,33,34]. A dense layer precursor solution was prepared by mixing 700 μL of titanium diiosopropoxide bis(acetylacetonate) in 7.0 mL of absolute ethanol and filtering through 0.2 μm pore syringe filter. 3.3 mL of the above solution was sprayed at 400 °C on to the cleaned FTO surface over a period of 10 min. and then annealed at 500 °C in a furnace for 10 min. It was then allowed to cool down to room temperature and ultra-sonicated in absolute ethanol for 20 min. The porous TiO_2_ thin film was deposited on the dense layer by spin coating a mixture of TiO_2_ paste (18 NR-T Dysol) and ethanol in 2:7 weight ratio (w/w) at 4500 rpm for 30 s. It was then calcinated at 450 °C for 30 min. The perovskite precursor solution was prepared by dissolving methylammonium iodide and lead (II) chloride in 3:1 molar ratio in 700 μL of DMF and stirring for 1 hr at 70 °C. This suspension was then spin coated onto the TiO_2_ porous layer at 2000 rpm for 30 s [35]. It was then sintered at 120 °C for 45 min. Then, selected HTMs were deposited by the following methods:

#### 2.2.1. CuI Powder Pressing

**Figure 1:**
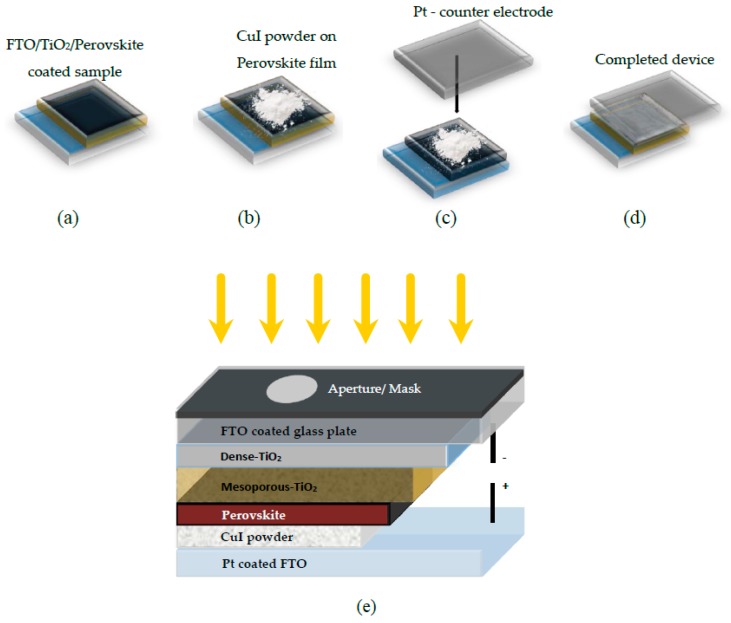
(**a**–**d**) step by step schematic of powder pressing method for incorporating cuprous iodide (CuI) powder as HTM in perovskite solar cells (PSCs) and (**e**) schematic representation of CuI device architecture.

Fine powder of CuI (98%, grain size of 0.2 µm) was spread onto the perovskite surface and the cell was completed by pressing it with Pt - counter electrode (made by sputtering process on FTO glass) as shown in the Figure 1.

#### 2.2.2. Deposition of Spiro-OMeTAD

1 mL of 97 mg/mL spiro-OMeTAD in chlorobenzene mixed with 30.2 µL of 175 mg/mL Bis(trifluoromethane) sulfonamide lithium salt in acetonitrile and 9.7 µL of solution consists of 46.6% of 4-tert-butylpyridine in acetonitrile. This mixed solution was spin coated on the perovskite film at 2000 rpm for 30 s [35]. Finally, 80 nm Au electrode was deposited on spiro-OMeTAD coated devices by thermal evaporation at a constant evaporation rate of 0.2 Å/s under 1 × 10^−6^ torr vacuum. Except for thermal evaporation, all the device fabrications were carried out in air. Figure 1e shows a schematic representation of CuI device architecture.

### 2.3. Electrical Characterization

The photovoltaic performance of fabricated PSCs was measured by using a computer controlled Keithley 2400 source meter (Cleveland, OH, USA) with a delay time of 50 ms under illuminations of 100 mW/cm^2^ (1 sun) with air mass (AM) 1.5 filter, with a solar simulator (Peccell-PEC-L12, Kanagawa, Japan). The solar simulator was calibrated using standard Silicon photodiode. The active area of the cell was 4.2 mm^2^. All samples were measured in air at room temperature.

### 2.4. Structural Characterization

The crystalline structure and phase purity of CuI powder were analyzed using X-ray diffraction spectroscopy (XRD, PANalytical-AERIS, Almelo, The Netherland). The diffraction pattern was collected with Cu Kα radiation (λ = 1.5408 Å) at ambient temperature, under the following operating conditions; accelerating voltage of 40 kV; emission current of 44 mA; scanned range (2*θ*) between 10°and 90° with a step size of 0.0027° and a scan speed of 4°/min.

## 3. Results and Discussion

**Figure 2:**
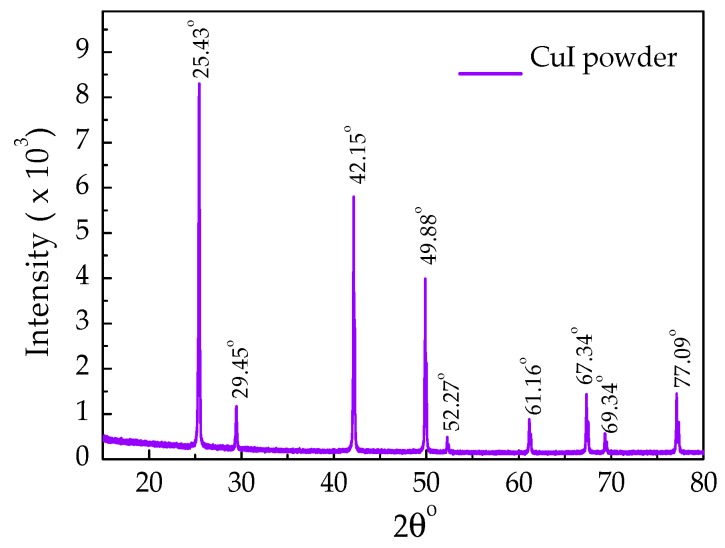
XRD spectra of Cuprous Iodide powder.

Firstly, CuI powder was characterized using XRD. Figure 2 shows the XRD pattern of as-received CuI powder, in which the grain size of 0.2 µm. The CuI powder exhibits major diffraction peaks at 2θ values of 25.43°, 29.45°, 42.15°, 49.88°, 67.34°, and 77.09° correspond to (111), (200), (220), (311), (331) and (422) planes, respectively. These are in good agreement with the structure of γ-CuI (JCPDS Card No. 06-0246) [20,36]. The higher peaks at 25.43° show that the orientation of γ-CuI is (111) preference [37]. However, a few additional peaks were also observed in the XRD pattern, which could be attributed to the fact of the reaction of CuI in air, moisture, and impurities.

**Figure 3:**
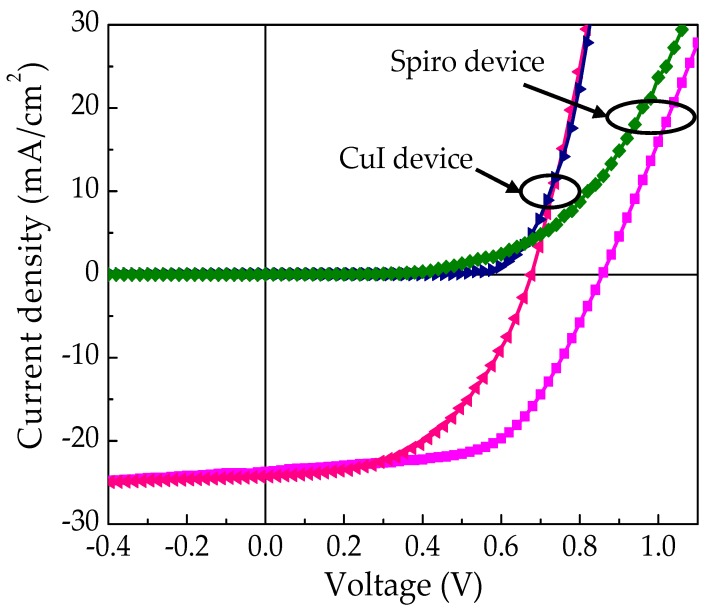
J-V characteristic of champion FTO/TiO_2_/MAPbI_x_Cl_3−x_/CuI/Pt and FTO/TiO_2_/MAPbI_x_Cl_3−x_/spiro-OMeTAD/Au devices under illuminations of 100 mW/cm^2^ (1 sun) with air mass (AM) 1.5 filter and in dark.

Then the opto-electronic properties of the fabricated PSCs were examined under 100 mW/cm^2^, AM 1.5 simulated solar irradiation (1 sun). The J-V characteristic of the best (champion) devices with spiro-OMeTAD and CuI as the HTM are shown in Figure 3. Table 1 summarizes the average photovoltaic properties of six solar cells fabricated with each of two hole-conducting material along with the performance of the champion cell. It is clear that the PV performance of the devices with spiro-OMeTAD as the HTM outperformed the CuI devices. As discussed in the literature, the spiro-OMeTAD is shown to be the best HTM for PSCs so far. However, it can be noted that the J_SC_ of the devices with CuI is marginally higher than that of the spiro devices fabricated under ideal conditions. As in Table 1, the average J_SC_ of the CuI devices was 24.09 ± 1.4 mA/cm^2^ which is higher than that of spiro-OMeTAD devices (22.4 ± 1.7 mA/cm^2^). This is consistent with higher hole-mobility found in CuI than spiro-OMeTAD to CuI [38]. The efficient charge extraction between the perovskite and CuI lead to an excellent hole injection from the active perovskite films to CuI layer [39].

However, the performance of the devices with CuI as HTMs was limited with lower V_OC_ (0.66 ± 0.02 V) and FF (0.49 ± 0.03) than that of the devices with spiro-OMeTAD (V_OC_ = 0.79 ± 0.03 V and FF = 0.56 ± 0.07) as HTMs. The lower fill factor of the CuI devices can be correlated to the higher thickness and roughness of the pressed CuI layer. Thickness of the CuI layer should be optimized to further enhance the efficiency of the cells [40]. According to our experience, controlling the thickness (~1 mm) of the CuI layer by using the pressing method is quite challenging. The average V_OC_ of the CuI based PSCs, were 0.66 ± 0.02 V, which is lower than spiro-OMeTAD based PSCs, that we tested under the same conditions. The lower V_OC_ of CuI devices can be attributed to an increase in recombination rate, which is due to induced valance band trap states caused by free iodine ions in the CuI [15]. It should be noted that the spiro-OMeTAD devices fabricated in our laboratory have efficiencies lower than that of reported spiro-OMeTAD devices in a closed environment [41]. In our work, all the device fabrication process and characterizations were done in an open atmosphere. This could be the reason for the lower performance of the devices.

**Figure 4:**
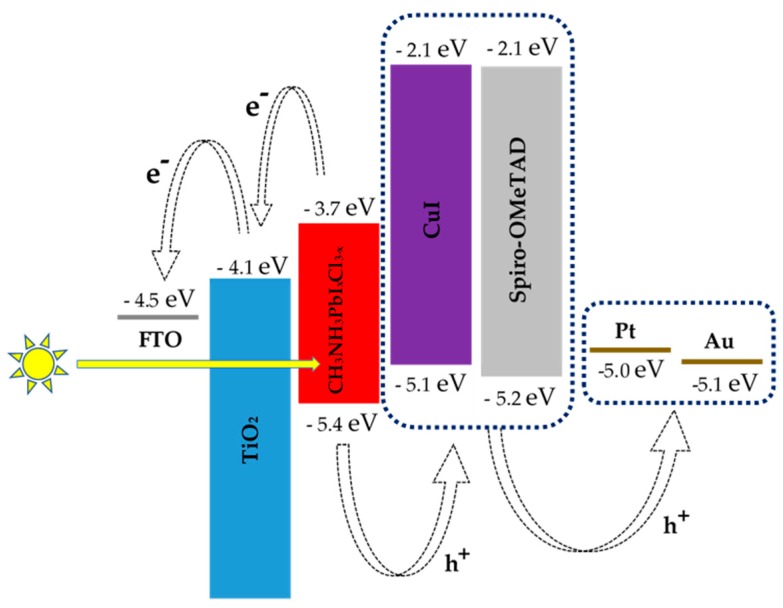
Proposed energy band diagram for the fabricated PSCs.

Figure 4 illustrates the proposed energy band diagram of fabricated PSCs in our work. The energies are expressed in electron volts (eV), using the electron energy in a vacuum as the reference. The conduction band (CB) of TiO_2_ and CH_3_NH_3_PbI_x_Cl_3−x_ are at −4.1 eV and −3.7 eV, respectively. Whereas, the edge of the valance band (VB) of CH_3_NH_3_PbI_x_Cl_3−x_ and CuI are at −5.4 eV and −5.1 eV respectively. The spiro-OMeTAD has HOMO levels around −5.2 eV [42,43,44,45]. It is well-known that the selection of HTMs on the device architectures and device fabrication process affects the PV performance of the devices [46]. Device characteristics of PSCs reported in the literature with CuI as HTMs employing different techniques are listed in Table 2. Here we note that the selected HTMs, CuI and spiro-OMeTAD have similar levels for hole transport and the changes in the performance of the devices may be attributed to different hole mobilities of CuI and spiro-OMeTAD.

As listed in Table 2, except thermally evaporated CuI/Cu based PSCs, the average PCE of our device is the highest among the reported works on CuI as HTM for PSCs. However, V_OC_ and FF are slightly lower than that of reported works. This may be due to the high level of porosity in the CuI layer fabricated using the pressing method compared to other methods such as spin coating, spray coating, evaporation method, etc. This limits the PCE of CuI based devices due to air voids and the trapping effect of charge carriers. This study reveals that the pressing method is a facile, hazard free and cost-effective method, which can be employed effectively for PSCs with CuI as HTM.

## 4. Conclusions

In this study, the focus has been on replacing the highly expensive spiro-OMeTAD that is used as hole transporting material in perovskite solar cells with a low-cost, dopant-free alternative. The study has resulted in successful fabrication of efficient PSCs with CuI hole transporting material employing a novel and simple powder pressing method. A variety of FTO/TiO_2_/MAPbI_x_Cl_3−x_/CuI/Pt and FTO/TiO_2_/MAPbI_x_Cl_3−x_/spiro-OMeTAD/Au are fabricated and the champion devices are compared. A powder pressed CuI offers J_SC_ over 24 mA/cm^2^, V_OC_ of 0.67 V, fill factor of 0.50, which yields promising efficiency of 8.1% in air for the PSC. Although spiro-OMeTAD based PSC had higher V_OC_ and FF, powder pressed CuI had higher J_SC_. Promising efficiencies obtained with a simple pressing method of CuI in an open environment shows the high potential of CuI as hole transporting material in PSCs.

## Figures and Tables

**Table 1:** The variation of photovoltaic parameters for the different HTM devices under illuminations of 100 mW/cm^2^ (1 sun) with air mass (AM) 1.5 filter. (The values in bold text are from the champion cell).

Hole Transporting Material (HTM)	J_SC_ (mA/cm^2^)	V_OC_ (V)	FF	PCE (ŋ%)
Spiro-OMeTAD	22.4 ± 1.7 **23.7**	0.79 ± 0.03 **0.85**	0.56 ± 0.07 **0.59**	9.9 ± 1.3 **11.9**
CuI	24.09 ± 1.4 **24.23**	0.66 ± 0.02 **0.67**	0.49 ± 0.03 **0.50**	7.8 ± 0.3 **8.1**

**Table 2:** Comparison of perovskite solar cells (PSCs) reported in the literature with CuI as the hole transporting material fabricated by different techniques.

Device Structure	CuI Deposition Method	Jsc (mA/cm^2^)	V_OC_ (V)	FF	PCE (ŋ%)	Reference
TiO_2_/CH_3_NH_3_PbI_3_/CuI	Solution pumping process	17.8	0.55	0.62	6.0	[15]
TiO_2_/CH_3_NH_3_PbI_x_Cl_3−x_/CuI	Spray coating method	22.3	0.61	0.42	5.8	[17]
TiO_2_/CH_3_NH_3_PbI_3_/CuI	Spin coating method	14.7	0.42	0.40	2.2	[47]
TiO_2_/CH_3_NH_3_PbI_3_/CuI	Doctor blading method	16.7	0.78	0.57	7.5	[19]
TiO_2_/CH_3_NH_3_PbI_3_/CuI	Gas-solid treatment	32.7	0.73	0.31	7.4	[22]
CuI/CH_3_NH_3_PbI_3_/PCBM	Doctor blading method	12.3	0.57	0.47	3.4	[48]
CuI/CsSnI_3_/C_60_/BCP	Thermal evaporation	8.94	0.36	0.54	2.1	[23]
TiO_2_/CH_3_NH_3_PbI_3_/CuI/Cu	Thermal evaporation	23.0	0.85	0.47	9.2	[30]
TiO_2_/CH_3_NH_3_PbI_x_Cl_3−x_/CuI	Powder pressing method	24.23	0.67	0.50	8.1	This work
